# Supplementary material for: Vaccination coverage and adverse events following a reactive vaccination campaign against hepatitis E in Bentiu displaced persons camp, South Sudan
Source: PLoS Negl Trop Dis. 2024 Jan 22;18(1):e0011661. doi: 10.1371/journal.pntd.0011661 (PMC10833508; doi:10.1371/journal.pntd.0011661)
Supplement: S1 Text — Table A. Vaccination coverage with exact number of doses according to recall and by card, n = 1599. Table B. Sensitivity analysis including only those who self-reported, n = 513. Table C. Sensitivity analysis of vaccination coverage by dose according to recall and card, n = 1470 individuals 16–39 yrs. (DOCX) [file pntd.0011661.s001.docx]

**Supporting Information**

**Table A.** Vaccination coverage with exact number of doses according to recall and by card, n=1599.

|  | **According to recall** | |  | **Confirmed by card** | |  |
| --- | --- | --- | --- | --- | --- | --- |
| **Coverage by dose** | **% (n)** | **95 % CI** | **DEFF** | **% (n)** | **95 % CI** | **DEFF** |
| None | 13.9% (222) | [12-16] | 1.6 | 59.7% (955) | [57-63] | 1.7 |
| One dose | 13.6% (217) | [12-16] | 1.3 | 21.2% (339) | [18.9-23.7] | 1.5 |
| Two doses | 14.8% (236) | [13-17] | 1.2 | 8.9% (142) | [7.5-10.5] | 1.2 |
| Three doses | 57.8% (924) | [55-61] | 1.7 | 10.2% (163) | [8.7-12.0] | 1.2 |
| Note that confirmed by card means that specific doses reported were verified on vaccination card. | | | | | | |

**Table B.** Sensitivity analysis including only those who self-reported, n=513.

|  | **According to recall or card** | |  | **Confirmed by card** | |  |
| --- | --- | --- | --- | --- | --- | --- |
| **Coverage by dose** | **% (n)** | **95 % CI** | **DEFF** | **% (n)** | **95 % CI** | **DEFF** |
| One or more doses | 89% (457) | [86-92] | 1.2 | 58% (296) | [53-57] | 1.2 |
| Two or more doses | 74% (378) | [69-78] | 1.4 | 32% (162) | [27-36] | 1.1 |
| Three doses | 58% (297) | [53-63] | 1.2 | 19% (96) | [15-22] | 1.2 |
| Note that confirmed by card means that all doses reported were verified on vaccination card. | | | | | | |

**Table C.** Sensitivity analysis vaccination coverage by dose according to recall and card, n=1470 individuals 16-39 yrs.

|  | **According to recall or card** | |  | **Confirmed by card** | |  |
| --- | --- | --- | --- | --- | --- | --- |
| **Coverage by dose** | **% (n)** | **95 % CI** | **DEFF** | **% (n)** | **95 % CI** | **DEFF** |
| One or more doses | 85% (1255) | [83-88] | 1.6 | 38% (558) | [35-41] | 1.7 |
| Two or more doses | 72% (1056) | [69-75] | 1.7 | 18% (258) | [15-20] | 1.2 |
| Three doses | 57% (845) | [54-61] | 1.7 | 9% (135) | [8-11] | 1.2 |
| Note that confirmed by card means that all doses reported were verified on vaccination card. | | | | | | |
