# Supplementary figures and images for: Vaccination coverage and adverse events following a reactive vaccination campaign against hepatitis E in Bentiu displaced persons camp, South Sudan
Source: PLoS Negl Trop Dis. 2024 Jan 22;18(1):e0011661. doi: 10.1371/journal.pntd.0011661 (PMC10833508; doi:10.1371/journal.pntd.0011661)

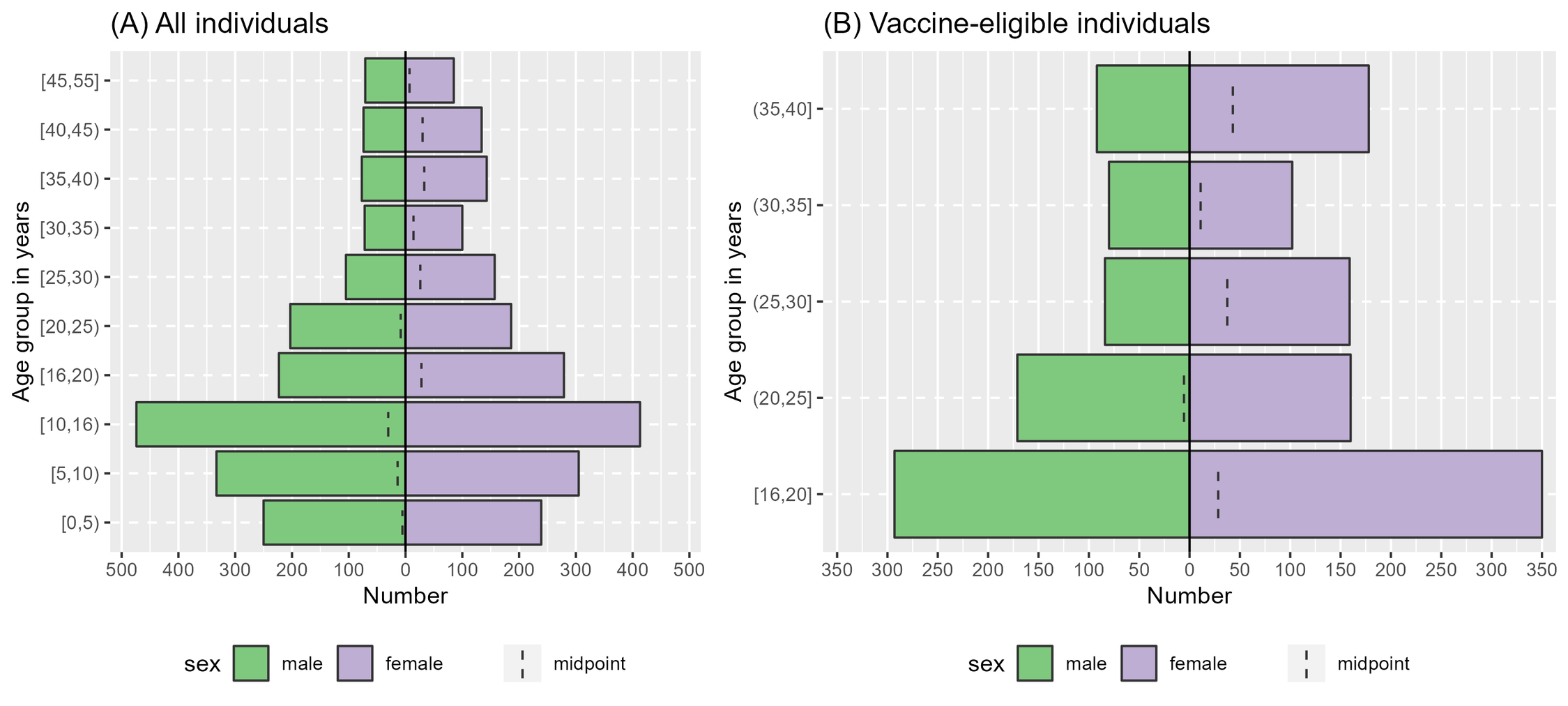

Supplement: S1 Fig — Population pyramid for all individuals counted at randomly selected households (A) and vaccine-eligible individuals counted at randomly selected households (B). (TIF) [file pntd.0011661.s002.tif]
