# Supplementary material for: Vaccination coverage and adverse events following a reactive vaccination campaign against hepatitis E in Bentiu displaced persons camp, South Sudan
Source: PLoS Negl Trop Dis. 2024 Jan 22;18(1):e0011661. doi: 10.1371/journal.pntd.0011661 (PMC10833508; doi:10.1371/journal.pntd.0011661)
Supplement: Attachment — Submitted filename: Response to reviewers 20231127.docx [file pntd.0011661.s003.docx]

**Reviewer #1:**

Reviewer #1: In the manuscript entitled “Vaccination coverage and adverse events following a reactive vaccination campaign against hepatitis E in Bentiu displaced persons camp, South Sudan”, Robin Nesbitt et al. reported the vaccination coverage and occurrence of AEFI through a sampling survey conducted in the context of mass hepatitis E vaccination campaigns in South Sudan. First of all, it must be acknowledged that the reactive vaccination campaign against hepatitis E in Bentiu internally displaced camp is an immensely commendable and challenging job. It not only would effectively reduce the incidence of hepatitis E in this high-prevalence area but also diminish the burden of severe illness and death caused by hepatitis E. Furthermore, it sets a precedent for future reactive vaccination campaigns in other regions. The author's investigation of vaccination coverage and AEFI conducted after this vaccination campaign is also of paramount importance. Firstly, having a relatively precise understanding of vaccination coverage provides a basis for exploring vaccine effectiveness in the future. Secondly, hepatitis E vaccine had not been widely used in high-prevalence areas or among pregnant women before this campaign, making this safety data particularly significant. Considering the challenges of conducting surveys in this highly mobile population, at this stage, my comments on this manuscript are as follows:

**Response**: We thank reviewer #1 for the detailed review of our results which have no doubt improved the clarity of our manuscript.

Major comments

1. Methods
   a. Line 95-100: It would be helpful if the authors could provide additional details regarding the sample size calculation, ideally including the formula used for the calculation. Additionally, explaining the meaning of design effect and how "deff=2" was determined, possibly with references, would help readers better understand the results of this study. And I haven’t found any explanation from the authors in either the results or discussion sections regarding the meaning of "Deff" in Tables 3 and 4.

**Response**: We calculated our sample size using the software ENA for SMART 2020, which is routinely used for sample size calculations for cross-sectional surveys. The formula for the sample size is the following:

$$n=\left[ t^{2} \times\frac{p \times q}{d^{2}} \right]\times DEFF$$

Where: n = sample size; t = z score for cluster sampling (2.045); p = expected prevalence (as fraction); q = 1 – p; d = relative desired precision; DEFF = Design effect.

We did not include the formula in the manuscript but have added a reference to the software.

DEFF is the design effect which is a measure that compares the sampling variance in the cluster survey sample to the sampling variance in survey using simple random sampling of the same size. For the sample size calculation, we estimated a value for the DEFF of 2, meaning that the cluster survey has two times as much variance as a survey using simple random sampling would have had. This is a conservative DEFF estimate that assumes that vaccination status within households (our clusters) will be similar. In the calculated DEFF values for one dose coverage we found DEFF estimates of 1.6 (recall and card) and 1.8 (card), which shows substantial household clustering of vaccination status. The possible harm of having estimated too high a DEFF in the sample size estimate is that we included too many households in the survey, which has logistical and financial cost, but would not negatively impact the precision of our estimates. We have added the term and acronym to the paper line 98 and in the foot note for tables 3 and 4. We have also added an interpretation of the design effect to the results on line 170.

b. Could the authors please clarify the definitions of “the target population” and “the vaccine-eligible population”, and the relationship between them in the Methods section? The sentence at line 100 “The target population for the survey was vaccine-eligible individuals” makes it confusing to read lines 162-166 of the Results. Which population's coverage is reported as the survey's result, and which is estimated based on the survey's findings?

**Response:** We have used these terms interchangeably. The target population for the vaccination campaign were individuals aged 16 to 40 years old residing in the Bentiu IDP camp, with no known contraindications, as defined on line 102-106. This is the “vaccine-eligible population”, 28,848 people targeted during the campaign. This is also the entire target population for whom the survey attempts to estimate vaccination coverage. We selected a sample of Bentiu IDP camp residents aged 16 to 40 years old to interview. The survey’s result estimates the vaccination coverage for the entire target population of vaccine-eligible individuals (16 to 40 years old residents of Bentiu IDP camp).

To clarify, we have replaced the use of ‘target population’ with the term ‘vaccine-eligible population’ the results section on line 167 and in the discussion 299.

2. Results
   a. Line 157-160: The authors presented survey results on “previous hepatitis E infection” in the results section. What is the purpose and significance of this survey? I didn’t find any relevant explanations in the Methods or Discussion.

**Response**: We present results on previous hepatitis E infection which was a question added to the same coverage survey with the objective to estimate the proportion of the camp population that had experienced jaundice or hepatitis E since the camp was established. We added a sentence on line 113-115 in the methods section about this question in the survey.

b. Line 219-225: any AE should be expressed as an incidence rate rather than a proportion among those who had at least one AE.

**Response**: We added the incidence rate per 100 as reported in table 6 to the text in the paragraph lines 225-230.

c. Table 6: the 'n' for systemic reactions is 140, which exceeds the 91 people reported for any symptom?

**Response**: The total number of systemic reactions was 140, counting more than one per individual as an individual may have reported more than one reaction. This is clearly indicated in the table title and as a footnote at the bottom of the table. Reactions were counted individually, rather than at least one symptom per person.

d. The authors should double-check all the data in this manuscript to ensure the presented data are correct. The multiple numbers in the text are quite confusing, for example, the several instances of the term "target", such as lines 86-88, line100, line 149, line 295 and those in Table 1, should be clarified by the authors that if these “target” refer to the same population (it seems not while the numbers before and after do not align consistently). If not, the authors should consider rewording and adding footnote of the tables to provide greater clarity.

**Response**: We have double checked all data and tables. We clarified the target population in table 1, it refers to the number of people who were estimated to be eligible for vaccination in the entire Bentiu IDP population and the number of people that the vaccination campaign aimed to vaccinate. It is explained in the introduction on line 76 (“the vaccination campaign targeted 26,848 residents of Bentiu IDP camp aged 16-40 years old, including pregnant women”). We added the word “population” to the in lines 87-89 to clarify that the administrative coverage is calculated as the total number of people vaccinated divided by the target population (26,848).

3. Discussion
   a. I recommend that the authors further expand on the limitations of this study, including but not limited to the retrospective survey method that may lead to recall bias, the lack of a precise temperature definition for fever in the survey results, and no control group who didn’t vaccinated with hepatitis E vaccine to compare the incidence of AE.

**Response**: We added the lack of precise temperature definition for fever and lack of control group to line 275 of the discussion. We also highlighted the limitation of the retrospective survey on line 287, as a reason for different estimates of adverse event incidence.

b. Line 284-286: The authors mentioned that “There were multiple vaccination campaigns conducted in Bentiu IDP camp in 2022, including for SARS-CoV-2, cholera and measles and it is possible that individuals reported vaccination for a different antigen.”. This could not only potentially lead to vaccinees confusing the source of adverse reactions but also the concurrent use of different vaccines in a short time frame (even on the same day, if possible?) might increase adverse reactions to vaccines. The authors should clarify if such a possibility exists.

**Response**: None of the vaccination campaigns occurred concurrently. In fact, the delay in the implementation of the hepatitis E vaccination campaign was due to the implementation of a cholera vaccination campaign at the start of the year. Its noteworthy that the cholera vaccine is an oral vaccine, and this would limit confusion with the hepatitis E vaccine. The SARS-CoV-2 vaccination was available at primary health facilities in between hepatitis E vaccination rounds and was not provided in a mass reactive campaign. The measles vaccination campaign targeted children and occurred in the period between the second round (April 2022) and third round (November 2022) of the hepatitis E campaign.

We are confident that the interviewers described in sufficient detail the specific vaccination campaign that we were assessing; they showed pictures of the vaccine (unique single dose presentation), the vaccination card received after vaccination (a unique green colour with MSF logo), they gave specific dates of the campaigns, and they also described the sites where people could go to get vaccinated.

The assessment of adverse events in the survey referred only to the hepatitis E vaccine received in October, directly before the survey, interviews took place a median of 23 days (range 3 – 36 days) after respondents were vaccinated in the third round.

Minor comments

1. There are several abbreviations in the main text without fully explanations. Please review the entire text and make the necessary revisions.

**Response**: Thank you for your detailed review, indeed we found several undefined abbreviations including MSF, IOM, ODK, PHCC, and ERB. We have defined all of these in the text.

2. Line 61: the vaccine efficacy of three doses of Hecolin over the first 4.5 years was 93.3% (95%CI 78.6–97.9). If the authors intend to present the VE of a single dose of hepatitis E vaccine, it is important to clearly specify "single-dose efficacy" in this sentence.

**Response**: Thank you for pointing out this oversight, we have clarified on line 61 that the long-term efficacy estimate we quote is from a modified intention to treat analysis of at least one dose over 54 months.

3. Line 112-114: repeated contents.

**Response**: We have deleted the repeated content.

4. Line 147: how to understand “abandoned”?

**Response**: We have added a definition to line 153 of abandoned as used by SMART methodology “unoccupied for a long time.”

5. Line 214: this subheading should be improved to be clearer and more understandable

**Response**: The subheading previously titled “survey” has now been updated to “Survey results on new symptoms within 72 hours of vaccination.” We hope this is clearer.

**Reviewer #2**

**Reviewer #2:** Use of this vaccine in an outbreak is long overdue and hopefully this will give confidence to other decision-makers to deploy this vaccine early in an outbreak and prevent unnecessary morbidity and mortality, especially among pregnant women. Other than the error on line 112, I believe this manuscript to be fully ready for publication.

**Response**: We thank reviewer #2 for supporting our work in advocating for the use of this vaccine in outbreaks and acknowledging that this change in practice could prevent unnecessary morbidity and mortality especially among pregnant women. We have deleted the repeated content from line 112.

**Reviewer #3**

Nesbitt et al in their manuscript titled “Vaccination coverage and adverse events following a reactive vaccination campaign against hepatitis E in Bentiu displaced persons camp, South Sudan” conducted a vaccination coverage survey on Hepatitis E using simple random sampling from the camp shelters following the third round of vaccination. They conducted the passive surveillance and reported the adverse events following immunisation (AEFI) on the reactive vaccine for peoples displaced in Bentiu IDP camp, South Sudan, and concluded that no severe AEFI were observed in people administered with hepatitis E vaccine of one to three doses in the Bentiu IDP camp community and suggested that the vaccine (Helicon) was accepted and well tolerated and should be considered for use in future outbreak response. Although the surveillance is on the relatively large-scale population with a sound conclusion and an useful information, I don’t believe that the contents is complete in term of reactive vaccination of Helicon in the population since the manuscript is only concerned with the safety. From this aspect, the manuscript should also include the efficacy aspect and therefore it is far from reaching the standard for this journal.

**Response**: We thank reviewer #3 for their comments, and interest in the vaccine efficacy estimates. As explained above we are unable to share vaccine effectiveness results at this time but hope to do so in the future. However, we think that the vaccination coverage and safety results merit publication on their own. This was the first time this vaccine was used in a mass reactive vaccination campaign, the high uptake, and few adverse events we document in this high-prevalence population sets a precedent that we hope will give confidence to governments to consider using the vaccine in future outbreaks. We refer the reviewer and editorial team to previously published articles on vaccine coverage following mass vaccination campaigns in this journal, including the first mass reactive vaccination campaign with the oral cholera vaccine published by some members of our team (1), use of the single-dose cholera vaccine several years later (2), and the delayed second dose more recently in PLoS One (3). We hope you and the reviewers will find that a revised version of this manuscript will reach the standard for this journal.

Minor corrections

1. Line 20: Hepatitis E (HEV) genotypes 1 and 2 are a common… change to “Hepatitis E (HEV) genotypes 1 and 2 are the common…”

**Response**: We have changed “a” to “the” online 20 of the abstract.

2. Line 233: …… found that the hepatitis E vaccine was highly accepted, we estimate that 86% (95% CI 84-88%) of the vaccine-eligible ….. change to “…… found that the hepatitis E vaccine was highly accepted. We estimate that 86% (95% CI 84-88%) of the vaccine-eligible …..”

**Response**: We have cut the sentence on line 233 into two sentences adding a period as suggested.

**References**

1.	Luquero FJ, Grout L, Ciglenecki I, Sakoba K, Traore B, Heile M, et al. First Outbreak Response Using an Oral Cholera Vaccine in Africa: Vaccine Coverage, Acceptability and Surveillance of Adverse Events, Guinea, 2012. PLoS Negl Trop Dis. 2013 Oct 17;7(10):e2465.

2.	Parker LA, Rumunu J, Jamet C, Kenyi Y, Lino RL, Wamala JF, et al. Neighborhood-targeted and case-triggered use of a single dose of oral cholera vaccine in an urban setting: Feasibility and vaccine coverage. PLoS Negl Trop Dis. 2017 Jun;11(6):e0005652.

3.	Ferreras E, Matapo B, Chizema-Kawesha E, Chewe O, Mzyece H, Blake A, et al. Delayed second dose of oral cholera vaccine administered before high-risk period for cholera transmission: Cholera control strategy in Lusaka, 2016. PloS One. 2019;14(8):e0219040.
